# A29 A NOVEL INTERACTIVE COMPLEX BETWEEN TP53, SIRT1 AND HNF4A2 IN INTESTINAL EPITHELIAL CELLS

**DOI:** 10.1093/jcag/gwae059.029

**Published:** 2025-02-10

**Authors:** J A Herrera Pulido, C Jones, F Boudreau

**Affiliations:** Universite de Sherbrooke, Sherbrooke, QC, Canada; Universite de Sherbrooke, Sherbrooke, QC, Canada; Universite de Sherbrooke, Sherbrooke, QC, Canada

## Abstract

NOT PUBLISHED AT THE AUTHOR’S REQUEST

Funding Agencies:

